# L-Tryptophan-Rich Diet Alleviates High-Intensity-Exercise-Induced Liver Dysfunction via the Metabolite Indole-3-Acetic Acid and AhR Activation

**DOI:** 10.3390/cells14080605

**Published:** 2025-04-16

**Authors:** Dawei Wang, Pengfei Hou, Hedong Lang, Yundong Xia, Qian Bai, Yu Yao, Long Yi, Mantian Mi

**Affiliations:** Research Center for Nutrition and Food Safety, Chongqing Key Laboratory of Nutrition and Health, Chongqing Medical Nutrition Research Center, Institute of Military Preventive Medicine, Third Military Medical University, Chongqing 400038, China; wang9610312022@163.com (D.W.); houpengfeilucky@tmmu.edu.cn (P.H.); lang0401@tmmu.edu.cn (H.L.); xyd@tmmu.edu.cn (Y.X.); baiqian0202@tmmu.edu.cn (Q.B.); yaoyuwork@163.com (Y.Y.); amu_yilong@tmmu.edu.cn (L.Y.)

**Keywords:** high-intensity exercise, liver, L-tryptophan, indole-3-acetic acid, aryl hydrocarbon receptor

## Abstract

High-intensity exercise (HIE) induces liver dysfunction and is detrimental to exercise performance. The underlying mechanism and preventive strategy urgently need to be explored. We increased the amount of tryptophan appropriately in the diet and explored the effect of an L-tryptophan-rich diet on the alleviation of HIE-induced liver dysfunction and the underlying mechanism. In this work, by establishing a C57BL/6 mouse model of high-intensity swimming exercise, the results demonstrated an L-tryptophan-rich diet significantly attenuated HIE-induced liver dysfunction, which was associated with increased levels of the tryptophan metabolite indole-3-acetic acid (IAA). Furthermore, IAA indeed exerted a protective effect against HIE-induced liver dysfunction in vivo and LPS-induced hepatocyte dysfunction in vitro. In conclusion, an L-tryptophan-rich diet may be a promising strategy to prevent HIE-induced liver dysfunction and metabolic disturbance via the metabolite indole-3-acetic acid and AhR activation.

## 1. Introduction

Several studies have reported that an appropriate amount of regular exercise benefits public health and prevents several chronic diseases, such as obesity, diabetes mellitus, and neurodegenerative disease [1]. However, high-intensity exercise (HIE) or excessive exercise, which refers to >60% maximal oxygen consumption (VO_2_ max) or >7.2 metabolic equivalents like marathons and triathlons, can result in tissue injuries and may be detrimental to public health [2,3,4]. Such stress could result in dysfunction, injury, and even disease of vital organs, particularly metabolic organs such as the liver. However, the underlying injury mechanisms and protective strategies remain unclear. The liver plays a central role in the management of systemic homeostasis via metabolic regulation processes such as glycogenolysis, gluconeogenesis, lipolysis, and ketogenesis [5]. Meanwhile, liver glycogen is an important substrate store and also represents a strong signal facilitating appropriate fuel selection to support prolonged endurance-type exercise. Liver injury induced by acute, prolonged, and high-intensity exercise has been well reported, characterized by fever, abdominal discomfort, and elevated serum ALT and AST levels [6]. Under HIE, the liver is prone to inflammatory damage, which can further cause systemic damage. The overproduction of inflammatory cytokines can contribute to the pathologic progression of damaged tissue. However, the metabolic landscape and mechanism of HIE-induced liver dysfunction as well as the protective strategies remain unknown.

Some studies have suggested that several nutrient metabolism disorders are closely related to liver dysfunction. For example, dietary lipids shape nonalcoholic steatohepatitis progression and infiltrating macrophages [7]. Branched-chain amino acids can reduce liver cell damage caused by lipid toxicity in rat liver cells with cirrhosis. Tryptophan, an essential amino acid, and its metabolites are beneficial for the inhibition of metabolic disorder, inflammatory injury, and oxidative stress in several organs [8]. Tryptophan supplementation substantially improves dextran sulfate sodium (DSS)-induced colitis, reduces the expression of pro-inflammatory factors, and increases the expression level of IL-22 [9]. Tryptophan is mainly metabolized in three main pathways: the kynurenine, 5-hydroxytryptamine, and indole pathways [10]. Indole and indole derivatives such as indole-3-pyruvic acid and indole-3-acetic acid (IAA) may act as ligands of AhR, which is widely expressed in a variety of lymphocytes and hepatocytes, and AhR is a ligand-activated transcription factor that mediates numerous cellular responses [11]. However, whether tryptophan metabolism is related to HIE-induced liver injury and its mechanisms has not been clarified.

Thus, by establishing the C57BL/6 mouse model of high-intensity swimming exercise, this study aims to clarify the role and mechanism of tryptophan metabolism in HIE-induced liver dysfunction and explore the effect of an L-tryptophan-rich diet in the alleviation of HIE-induced liver dysfunction and its underlying mechanism.

## 2. Materials and Methods

### 2.1. Reagents

A control diet (AIN-93M) and an experimental diet with added tryptophan at a final dose of 0.5% were purchased from Jiangsu Xietong Pharmaceutical Bio-engineering Co., Ltd. (Nanjing, Jiangsu, China). CH223191 and IAA were obtained from Selleck (Houston, TX, USA). LPS was purchased from Sigma Aldrich (Saint Louis, MO, USA). The Cell Counting Kit-8 was obtained from Beyotime Biotechnology (Shanghai, China). ELISA kits were obtained from Shanghai Enzyme-linked Biotechnology Co., Ltd. (Shanghai, China). The MDA, CAT, SOD, and GSH assay kits were provided by Solarbio Science & Technology (Beijing, China).

### 2.2. Animals and Experimental Protocol

Male C57BL/6 mice weighing 18–20 g at 7 weeks of age obtained from Hunan Sja Laboratory Co., Ltd (Changsha, Hunan, China). were housed and bred in a controlled environment with a temperature of 22–25 °C and humidity of 50 ± 5% on a 12 h light–dark cycle. Food and water were changed every 3 days and were provided ad libitum.

#### 2.2.1. Experiment #1

Mice were randomly divided into two groups (n = 10/group): the control (CON) group and high-intensity exercise (HIE) group. The swimming exercise was carried out by every ten mice per plastic box (90 cm × 50 cm × 40 cm) filled with a 30 cm depth of water maintained at 34 ± 1 °C according to a previous report [12]. Briefly, mice were subjected to an adaptive training exercise for five days. Day 1, two 30 s swimming sessions with a 2 min rest interval between the swimming periods; Day 2, two 2 min swimming sessions with a 2 min rest interval; Day 3, three 10 min swimming sessions with a 5 min rest interval; Day 4, two 15 min swimming sessions with a 5 min rest interval; Day 5, a period of 30 min of swimming with no pause. After the adaptive training, mice in the CON group were kept in a box with a 2 cm depth of water for 2.5 h per day for seven days (fake exercise), while mice in the HIE group were kept in a box with a 30 cm depth of water and subjected to swimming for 2.5 h per day for seven days [13]. Day 6 and Day 7 were rest days.

#### 2.2.2. Experiment #2

To investigate the effect of L-tryptophan supplementation on HIE-induced liver injury, mice were randomly divided into three groups (n = 8/group): the CON, HIE, and HIE + TRP5 groups. The CON and HIE group mice were fed a control diet, which contained 0.16% tryptophan. And HIE + TRP5 group mice were fed a control diet with tryptophan supplementation at a final dose of 0.5%. Mice were subjected to adaptive training and HIE according to Experiment #1.

#### 2.2.3. Experiment #3

To investigate whether the protective effect of tryptophan on HIE-induced liver injury was mediated via AhR, mice were randomly divided into four groups (n = 8/group), HIE, HIE + TRP5, HIE + CH223191, and HIE + TRP5 + CH223191, respectively. Mice were subjected to adaptive training and HIE according to Experiment #1. And mice from the HIE and HIE + Trp5 groups were fed a control diet and a control diet with tryptophan supplementation according to Experiment #2. Mice were intraperitoneally administered 10 mg/kg of CH223191 (S7711, Selleck, Houston, TX, USA) or the vehicle (the same volume of corn oil) once per day from Day 7 to 14.

#### 2.2.4. Experiment #4

To investigate whether the protective effect of tryptophan on HIE-induced liver injury was mediated through its metabolite IAA, mice were randomly divided into two groups (n = 8/group), HIE and HIE + IAA, respectively. Mice were subjected to adaptive training and HIE according to Experiment #2. And mice were administered 50 mg/kg of IAA (Selleck, S4799, USA, QD, IP) or the vehicle (the same volume of corn oil) from Day 7 to 14.

Mice from each group were sacrificed immediately after the experiment, and the liver tissues and serum were harvested and stored at −80 °C. All animal experiments were approved by the Institutional Animal Care and Use Committees of Third Military Medical University (Chongqing, China; Approval SYXC-2015-00169) and followed by the National Research Council Guidelines.

#### 2.2.5. Study Design Subsection

To better understand the four experimental designs, a schematic diagram of the experimental designs was made. Figure A demonstrates the design of experiments 1–4. the interventions of the CON and HIE groups are outlined in Figure B (Supplementary Figure S2).

### 2.3. Non-Targeted Metabolomics and Targeted Tryptophan Metabolites Assay

The non-targeted metabolomics of liver tissue (100 μg) and the targeted tryptophan metabolite assay of serum (100 μL) were performed by Shanghai Applied Protein Technology Co., Ltd., Shanghai, China.

### 2.4. Metabolomics Data Processing and Analysis

After sum-normalization, the processed data were analyzed using R package (3.3.5, ropls), where it was subjected to multivariate data analysis, including Pareto-scaled principal component analysis (PCA) and orthogonal partial least-squares discriminant analysis (OPLS-DA). Seven-fold cross-validation and response permutation testing was used to evaluate the robustness of the model. The variable importance in the projection (VIP) value of each variable in the OPLS-DA model was calculated to indicate its contribution to the classification. Student’s *t*-test was applied to determine the significance of differences between two groups of independent samples. VIP > 1 and *p* value < 0.05 were used to screen significantly changed metabolites. Pearson’s correlation or Spearman’s correlation analysis was performed to determine the correlation between two variables.

### 2.5. Cell Culture and Treatment

HepG2 cells were archived in our laboratory and cultured in DMEM supplemented with 10% FBS. Cells were incubated at 37 °C in a humidified atmosphere containing 5% CO_2_. After reaching 70% confluency, cells were stimulated with IAA (0.25, 0.5, and 1 mM) for 12 h, followed by treatment with or without 10 ng/mL of LPS (HY-D1056, Sigma Aldrich, Saint Louis, MS, USA) for an additional 12 h in serum-free media containing 25 mM of glucose and 0.25% BSA. Cell viability was measured using Cell Counting Kit-8 (C0042, Beyotime, shanghai, China).

### 2.6. ALT and AST Measurements

The serum was prepared by solidification and centrifugation (4 °C, 3000× *g*, 10 min) and then stored at −80 °C. Biochemical measurements of aspartate aminotransferase (AST) and alanine aminotransferase (ALT) were performed on an Olympus AV5400 auto analyzer(Olympus, Tokyo, Japan). Additionally, AST in cell supernatant was measured using a Micro Glutamic-oxalacetic Transaminase Assay Kit (BC1565, solarbio, Beijing, China).

### 2.7. Enzyme-Linked Immunosorbent Assay

The mouse serum and HepG2 cell culture supernatant were quantified using a tumor necrosis factor (TNF)-α ELISA kit (ml002095-C, MIbio, Shanghai, China) according to the manufacturer’s instructions. A SpectraMax^®^ M2 spectrophotometer (Molecular Devices Corp., Waltham, MA, USA) was used to obtain the OD value at 450 nm.

### 2.8. Determination of Hepatic Parameters

The level of malonaldehyde (MDA, BC0025, Solarbio Science & Technology, Beijing, China) and the activity of superoxide dismutase (SOD, BC0175, Solarbio Science & Technology, Beijing, China), catalase (CAT, BC0205, Solarbio Science & Technology, Beijing, China), and reduced glutathione (GSH, BC1175, Solarbio Science & Technology, Beijing, China) were measured using the corresponding commercial assay kits according to the manufacturer’s instructions, respectively. A SpectraMax^®^ M2 spectrophotometer (Molecular Devices Corp., Waltham, MA, USA) was used to obtain the OD value at 450 nm.

### 2.9. Quantitative Real-Time Polymerase Chain Reaction (qRT-PCR)

The total RNA was extracted with TRIzol reagent (#9019, Takara, Tokyo, Japan). We used a qTower 2.2 real-time PCR system (Analytik Jena, Berlin, Germany) to run qRT-PCR with SYBR Premix Ex Taq II (#RR820A, Takara Bio, Tokyo, Japan). All primers are listed in Table 1. The relative mRNA expression levels of the targeted genes were normalized to that of β-actin and calculated using the 2^−ΔΔCt^ method.

### 2.10. Western Blot Analysis

The protein expression was investigated through Western blot analysis according to the previous method [14]. The corresponding primary antibodies were used to analyze the protein expressions of NF-κB p65 (#8242; Cell Signaling Technology, BOS, MA, USA), phospho-NF-κB p65 (#3033; Cell Signaling Technology, BOS, MA, USA), IκBα (#4814; Cell Signaling Technology, BOS, MA, USA), and β-actin (#47778, Cell Signaling Technology, BOS, MA, USA), respectively.

### 2.11. Histological Staining and Immunofluorescence Analysis

After animals were sacrificed, liver samples were fixed with 4% paraformaldehyde and embedded in paraffin immediately. Hematoxylin and eosin (H&E) and PAS staining were performed with the liver sections. The sections were scanned using a high-resolution digital slide scanner (VS-200, Olympus, Tokyo, Japan). To detect the macrophages in the liver, the sections were stained with an anti-F4/80 rabbit pAb (GB11027, Servicebio, Beijing, China). The images were collected using fluorescent microscopy (Nikon Eclipse C1, Nikon, Tokyo, Japan). The number of F4/80^+^ cells was calculated from three high-power fields (HPF, ×400 magnification) per mouse.

### 2.12. Detection of Intracellular Reactive Oxygen Species (ROS)

Two kinds of fluorescent probes, ROS staining solution (SIGMA, D7008, Saint Louis, MS, USA) and dihydroethidium (DHE, E004-1-1, Nanjing Jiancheng Bioengineering Institute, Nanjing, China), were used to detect the intracellular ROS level in liver tissues and HepG2 cells, respectively. For the ROS staining, after restoring frozen slides to room temperature, the spontaneous fluorescence quenching reagent was added for 5 min. Then, the cryosections were incubated with DAPI solution at room temperature for 10 min in the dark. For the DHE staining, cryosections were incubated with 10 μM of DHE at 37 °C for 30 min. The SpectraMax^®^ M2 spectrophotometer (Molecular Devices Corp., Waltham, MA, USA) was used to obtain the OD value at a 488 nm excitation wavelength and 525 nm emission wavelength. The images were collected using fluorescent microscopy (Nikon Eclipse C1, Nikon, Japan). The mean density was calculated from three high-power fields (HPF, ×200 magnification) per mouse.

### 2.13. Molecular Docking

The protein coordinates (PDB: 8H77), downloaded from the Protein Data Bank (http://www.rcsb.org/pdb/, 12 February 2023), were chosen as templates. Molecular docking calculations were conducted using the Dock 6.9 protocol in Yin fu Cloud Platform (http://cloud.yinfotek.com/, 26 February 2023). The binding pocket of the crystal ligand was set according to a previous study [15] and then the Grid-based score was calculated for the pose.

### 2.14. Statistical Analysis

Data analysis was performed with GraphPad Prism 6.0 (GraphPad Software, Inc., La Jolla, CA, USA). All experimental data were expressed as mean ± SEM. Differences between groups were determined using Student’s *t*-test (two groups) or one-way analysis of variance (ANOVA) followed by Dunnett’s test or Tukey’s test (multiple groups) according to the normality test (Shapiro–Wilk normality test). Pearson’s correlation coefficient analysis or Spearman’s correlation coefficient analysis was used for correlation analysis. P-values less than 0.05 were considered statistically significant. All experiments were repeated at least three times.

## 3. Results

### 3.1. HIE Induced Aggravated Liver Dysfunction and Metabolic Disturbance

**Figure 1:**
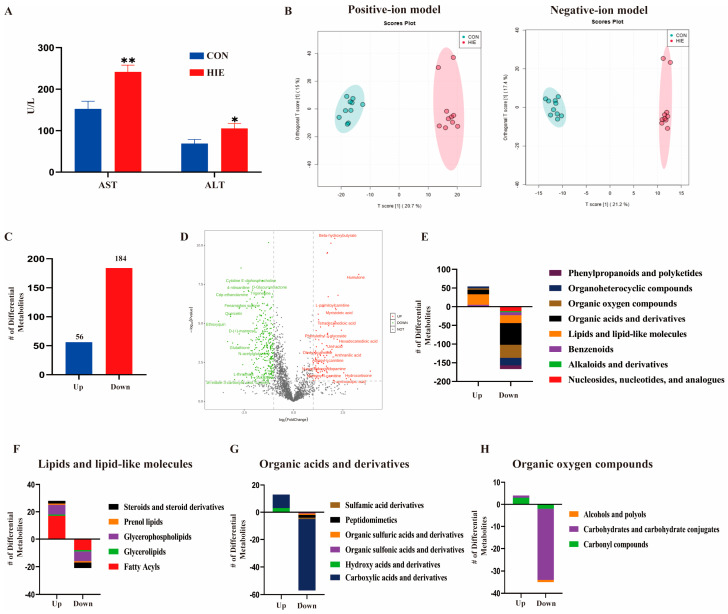
HIE induced aggravated liver dysfunction and metabolic disturbance. (**A**) Serum concentrations of ALT and AST (n = 6). (**B**–**H**) Non-targeted metabonomics derived from LC-MS/MS metabolite profiles of liver tissue from mice in the CON group (n = 10) and HIE group (n = 10), OPLSDA plots in positive-ion or negative-ion mode of mice livers (**B**), of which green dots represent the CON group and pink dots the HIE group. The numbers of different metabolites that were up- or downregulated in the comparison (**C**) and volcano plots of different metabolites (**D**) are displayed. The different metabolites can be categorized into different classes (**E**), including lipids and lipid-like molecules (**F**), organic acids and derivatives (**G**), and organic oxygen compounds (**H**). Data were analyzed using an independent sample Student’s *t*-test (**A**). Error bars (**A**) show SEM. * *p* < 0.05, ** *p* < 0.01, compared to the control group. *p*-values less than 0.05 were considered statistically significant.

To observe the effect of HIE on liver injury, mice were randomly assigned to the control and HIE groups. Compared with the control group, the serum ALT and AST levels in the HIE group were significantly increased (Figure 1A). The OPLSDA revealed that HIE had a notable effect on the metabolome in liver tissues by the positive and negative ion models (Figure 1B). Furthermore, there were a total of 240 identified differential metabolites, of which 184 were decreased and 56 were increased in the HIE group (Figure 1C). A volcano diagram is shown in Figure 1D. According to these changes in metabolite class, organic acids and derivatives as well as the organic oxygen compounds were decreased while the lipids and lipid-like molecules were increased followed by HIE, compared with the control group (Figure 1E). According to metabolite subclasses, the lipids and lipid-like molecules included fatty acyls, glycerophospholipids, steroids, and steroid derivatives (Figure 1F). The organic acids and derivatives mainly included carboxylic acids and derivatives, hydroxy acids and derivatives, peptidomimetics, and organic sulfuric acids and derivatives (Figure 1G). Additionally, the organic oxygen compounds contained carbohydrates and carbohydrate conjugates as well as carbonyl compounds (Figure 1H). As shown in Table 2, in the top differential metabolites among the three classes, beta-hydroxybutyrate, L-palmitoylcarnitine, myristoleic acid, tetradecanedioic acid, and humulone were increased, while D-Glucuronate, L-Sorbose, maltotetraose, lactulose, isomaltose, malonic acid, pro-hyp, and histidine were decreased following HIE. Taken together, the results indicate that HIE induces aggravated liver dysfunction and metabolic disturbance.

### 3.2. HIE-Induced Tryptophan Metabolic Disorder in Liver, Serum and L-Tryptophan-Rich Diet Ameliorated HIE-Induced Liver Dysfunction

**Figure 2:**
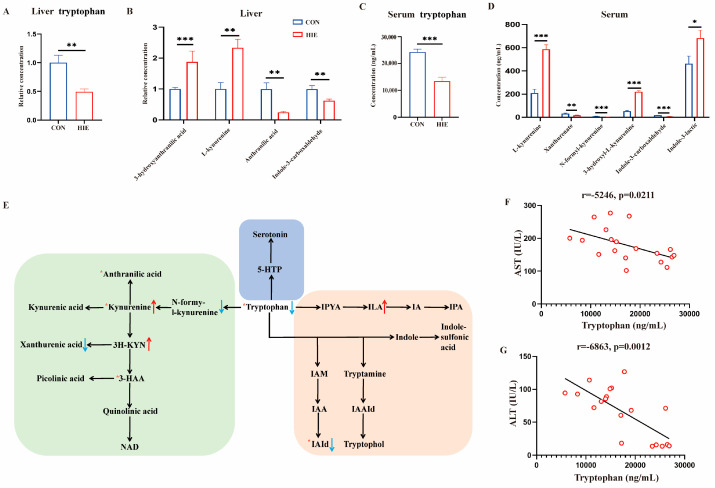
HIE-induced tryptophan metabolic disorder in the liver and serum. (**A**,**B**) Untargeted metabolomic detection of hepatic tryptophan and its metabolite concentrations (n = 10). (**C**,**D**) The concentration of serum tryptophan and its metabolites detected via targeted metabonomics (n = 8). (**E**) Metabolic pathway diagram of tryptophan in the liver and serum (red arrow, up in serum; blue arrow, down in serum; red asterisk, changes in liver). Correlation analysis between serum tryptophan content and ALT (**F**) and AST (**G**) in mice (n = 19). Data were analyzed using the independent sample Student’s *t*-test (**A**–**D**). Error bars (**A**–**D**) show SEM. * *p* < 0.05, ** *p* < 0.01, *** *p* < 0.001, compared to the control group. *p*-values less than 0.05 were considered statistically significant. Pearson’s or Spearman’s correlation analysis was used for correlation analysis.

**Figure 3:**
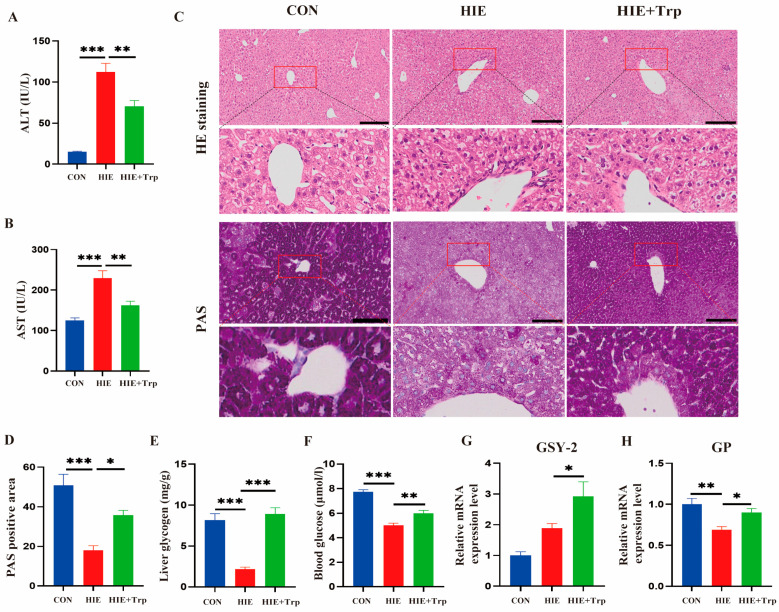
L-tryptophan-rich diet ameliorated HIE-induced liver dysfunction. (**A**,**B**) Serum concentrations of ALT and AST (n = 6). (**C**) H&E staining of liver. Magnification ×100; scale bars, 200 μm. Liver glycogen shown through PAS staining. Magnification, ×200; scale bars, 200 μm. (**D**) Statistical analysis of the PAS-positive area (n = 4). (**E**) Glycogen level in the liver of mice (n = 7). (**F**) Blood glucose level (n = 8). (**G**,**H**) The mRNA expression of GSY-2 and GP in the liver of mice using qRT-PCR (n = 5). Data were analyzed using ANOVA test (**B**–**H**). Error bars (**B**–**H**) show SEM. * *p* < 0.05, ** *p* < 0.01, *** *p* < 0.001, compared to the control group or HIE group. *p*-values less than 0.05 were considered statistically significant.

Firstly, according to the liver metabolome, we found that the tryptophan level in the liver was significantly decreased and the levels of tryptophan metabolites such as 3-hydroxyanthranilic acid, L-kynurenine, anthranilic acid, and indole-3-carboxyaldehyde were also changed (Figure 2A,B). Furthermore, the results of the serum targeted metabolome demonstrated that the serum tryptophan level was decreased, while tryptophan metabolites such as L-kynurenine, xanthurenate, N-formyl-kynurenine, 3-hydroxyl-kynurenine, indole-3-carboxyaldehyde, and indole-3-lactic were significantly changed (Figure 2C,D). A schematic of tryptophan metabolism of the liver and serum is showed in Figure 2E. Correlation analysis indicated that the serum tryptophan level was significantly negatively correlated with the serum AST (r = −0.5246, *p* = 0.0211) and ALT (r = −0.6863, *p* = 0.0012) (Figure 2F,G). Based on the results, mice were subjected to HIE with or without L-tryptophan supplementation. An L-tryptophan-rich diet improved liver injury, as indicated by the decreased serum ALT and AST levels (Figure 3A,B) and H&E staining (Figure 3C) compared to the HIE group. Moreover, the liver glycogen (Figure 3C–E) and blood glucose levels (Figure 3F) as well as the mRNA levels of glycogen phosphorylase (GP) and glycogen synthase (GYS)2 (Figure 3G,H) were also increased in the L-tryptophan administration group, respectively. Thus, HIE-induced tryptophan metabolic disorder in the liver, serum, and L-tryptophan-rich diet ameliorated HIE-induced liver dysfunction and glycogen metabolism.

### 3.3. L-Tryptophan-Rich Diet Significantly Reduced HIE-Induced Liver Oxidative Stress and Inflammatory Response

**Figure 4:**
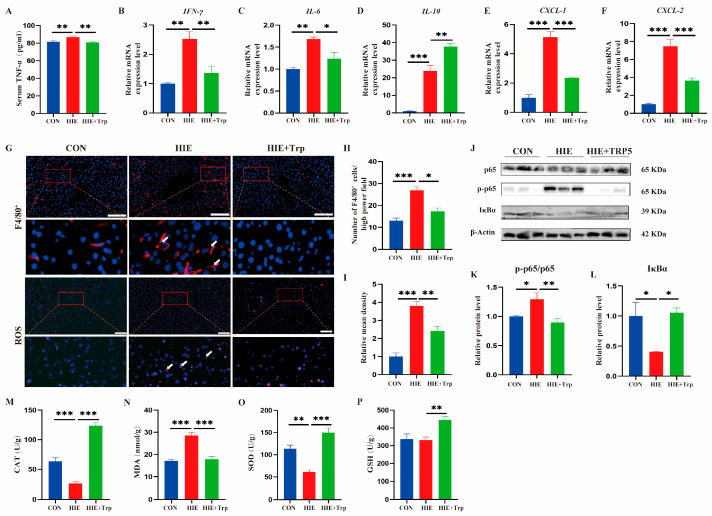
L-tryptophan-rich diet significantly reduced HIE-induced liver oxidative stress and inflammatory response. (**A**) Serum concentration of TNF-α protein in mice. (**B**–**F**) mRNA expression of IFN-γ (**B**), IL-6 (**C**), IL-10 (**D**), CXCL-1 (**E**), and CXCL-2 (**F**) in the liver of mice, as shown by qRT-PCR (n = 4). (**G**–**I**) Immunofluorescence staining of F4/80^+^ macrophages (**G**) and statistical analysis of the number of F4/80^+^ macrophages per high-power field (n = 4) (**H**). F4/80+ macrophages, red with white arrow; nuclei, blue. Magnification, ×200; scale bars, 100 μm. Fluorescence staining of ROS production in liver sections (ROS, red with white arrow; nuclei, blue). Magnification ×200; scale bars, 100 μm. (**I**) Quantification of ROS fluorescence intensity (n = 4). (**J**–**L**) Expression levels of the p65, p-p65, and IκBα proteins were assessed using Western blot (n = 4). (**K**) Relative ratio of p-p65 to p65 (n = 4). (**L**) Quantification of IκBα (n = 4). (**M**–**P**) CAT (**M**), MDA (**N**), SOD (**O**), and GSH (**P**) levels in mouse livers (n = 5). Data were analyzed using ANOVA test (**A**–**F**,**H**,**I**,**K**–**P**). Error bars (**A**–**F**,**H**,**I**,**K**–**P**) show SEM. * *p* < 0.05, ** *p* < 0.01, *** *p* < 0.001, compared to the control group or HIE group. *p*-values less than 0.05 were considered statistically significant.

To determine the effect of L-tryptophan supplementation on HIE-induced liver inflammation and oxidative stress, the mRNA expression in the liver and serum protein level of the inflammatory cytokines were detected. The results showed that the serum TNF-α was significantly decreased following L-tryptophan supplementation compared to the HIE group (Figure 4A). And the mRNA expression of pro-inflammatory cytokines IL-6 and IFN-γ was suppressed, while the mRNA expression of the anti-inflammatory cytokine IL-10 was increased via L-tryptophan intervention compared with the HIE group (Figure 4B–D). Meanwhile, we also found that the mRNA expression of chemokines CXCL-1 and CXCL-2 and the quantity of F4/80^+^ macrophages were significantly reduced (Figure 4E–H). Furthermore, the ratio of phospho-p65 to p65 was significantly reduced, while the protein expression levels of IκBα were increased significantly in the HIE+TRP5 group (Figure 4J–L). Additionally, the ROS (Figure 4G,I) and oxidative-stress-related enzymes including CAT, MDA, GSH, and SOD (Figure 4M–P) were detected in liver tissue, respectively. The indicators were notably reversed by L-tryptophan supplementation. These results indicate that an L-tryptophan-rich diet ameliorates liver dysfunction in response to HIE subjection, associated with the amelioration of liver inflammation and oxidative stress.

### 3.4. L-Tryptophan-Rich Diet Improved HIE-Induced Liver Dysfunction Associated with the Increased Tryptophan Metabolite IAA and AhR Activation

**Figure 5:**
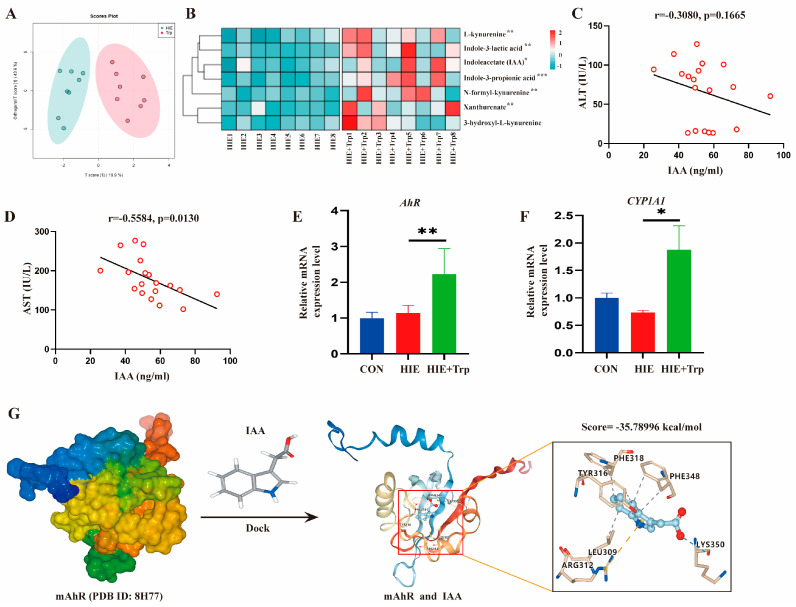
L-tryptophan-rich diet improved HIE-induced liver dysfunction associated with increased tryptophan metabolite IAA and AhR activation. (**A**) OPLSDA plots of mouse serum detected by targeted tryptophan metabolomics (n = 8). (**B**) Cluster heat map of tryptophan metabolites in mouse serum. Pearson’s or Spearman’s correlation analysis between IAA content and ALT (**C**) and AST (**D**) in mouse serum (n = 19). The mRNA expression of AhR (**E**) and CYP1A1 (**F**) in the liver of mice using qRT-PCR (n = 5–6). (**G**) Molecular docking of mAhR and IAA. Data were analyzed using ANOVA test (**E**,**F**). Error bars (**E**,**F**) show SEM. * *p* < 0.05, ** *p* < 0.01, *** *p* < 0.001, compared to the control group or HIE group. *p*-values less than 0.05 were considered statistically significant.

To explore how L-tryptophan supplementation improved HIE-induced liver dysfunction, we have measured the concentrations of 17 serum tryptophan metabolites in mouse serum through targeted tryptophan metabolite assay (Figure S1). The OPLSDA revealed that L-tryptophan supplementation had a notable effect on the target tryptophan metabolome in serum compared to the HIE group mice (Figure 5A). IAA (Figure S1A), Indole-3-lactic acid (Figure S1B), IPA (Figure S1C), L-kynurenine (Figure S1G), 3-hydroxyl-L-kynurenine (Figure S1M), Xanthurenate (Figure S1O), and N-formyl-kynurenine (Figure S1Q) were significantly increased in the L-tryptophan supplementation group compared to the group with a control diet in HIE mice (Figure 5B). However, the other metabolites (Figure S1D–F,H–L,N,P) remained statistically unchanged. Correlation analysis of the seven differential metabolites and serum AST or ALT was performed (Table 3), respectively. Interestingly, only one metabolite IAA was negatively correlated with the AST level (Figure 5C,D). Furthermore, as we know, indole and indole derivatives act as ligands of AhR; to observe the AhR activation, we analyzed the mRNA expression of liver AhR and CYP1A1, which is an AhR activation marker. AhR and CYP1A1 expressions were significantly upregulated through L-tryptophan treatment compared to the HIE group mice (Figure 5E,F). Next, molecular virtual docking validation was performed to examine the potential interactions between IAA and mouse AhR crystal structure (PDB ID:8H77), which could further show the binding conformation and detailed interaction information. We found that IAA formed a hydrogen bond interaction with the residue LYS350 and hydrophobic interactions with the residues PHE318, PHE348, TYR316, and LEU309 as well as the Π-cation interaction with the residue ARG312 (Figure 5G). The results indicate that an L-tryptophan-rich diet improves HIE-induced dysfunction and disturbed homeostasis associated with increased tryptophan metabolite IAA and AhR activation.

### 3.5. The Effect of an L-Tryptophan-Rich Diet on Improving HIE-Induced Liver Dysfunction Was Also Abrogated by the AhR Inhibitor CH223191

**Figure 6:**
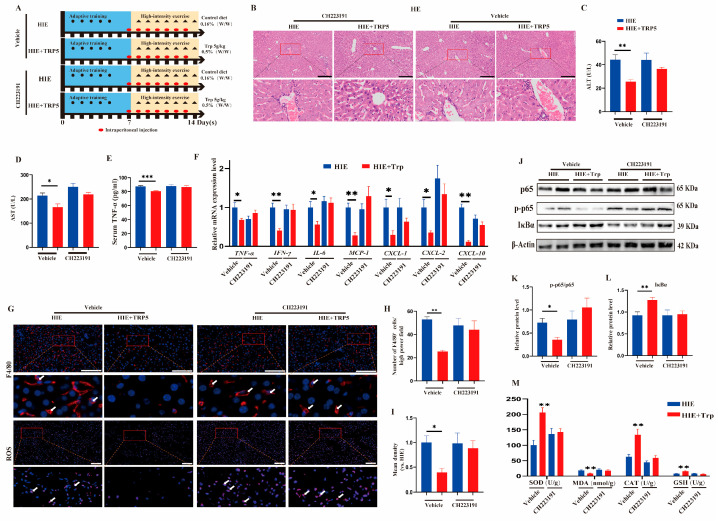
The effect of L-tryptophan-rich diet on improving HIE-induced liver dysfunction was also abrogated by the AhR inhibitor CH223191. (**A**) Schematic diagram of experimental design. Mice in the HIE and HIE + TRP5 groups were treated with DMSO (vehicle) or drug CH223191 from Day 7 to Day 13 consecutively for a total of 7 doses, 8 mice per group. The body weight and blood glucose of mice were assessed after exercise on the last day, and the mice were then sacrificed (black dots, adaptive training; triangle, HIE; red ellipse, intraperitoneal injection). (**B**) Histology (HE staining) of the liver of mice in the HIE and HIE + TRP5 groups treated with vehicle or CH223191. Magnification ×100. Scale bars, 200 μm. (**C**,**D**) Serum concentrations of ALT (**C**) and AST (**D**) of mice in the HIE and HIE + TRP5 groups treated with vehicle or CH223191 (n = 7). (**E**) Serum concentration of TNF-α of mice in the HIE and HIE + TRP5 groups treated with vehicle or CH223191 (n = 6). (**F**) The mRNA expression of MCP-1, CXCL-1, CXCL-2, IFN-γ, CXCL-10, TNF-α, and IL-6 in the liver of mice in the HIE and HIE + TRP5 groups treated with vehicle or CH223191 analyzed using qRT-PCR (n = 5). (**G**) Immunofluorescence staining of macrophages in liver sections of mice in the HIE and HIE + TRP5 groups treated with vehicle or CH223191. F4/80+ macrophages, red with white arrow; nuclei, blue. Magnification, ×200; immunofluorescence staining of ROS production (ROS, red with white arrow; nuclei, blue) in liver sections from mice in HIE and HIE + TRP5 groups treated with vehicle or CH223191. Magnification ×200. Scale bars, 100 μm. (**H**) Quantification of macrophages per high-power field (n = 4). (**I**) Quantification of ROS immunofluorescence intensity (n = 4). (**J**) The expression levels of p65, p-p65, and IκBα protein in the liver of mice in the HIE and HIE + TRP5 groups treated with vehicle or CH223191 assessed using Western blot. (**K**) The ratio of p-p65 to p65 (n = 4). (**L**) Quantification of IκBα/β-actin (n = 5). (**M**) Levels of CAT, SOD, MDA, and GSH in the liver of mice (n = 7). Data were representative of two independent experiments with at least five mice per group and were analyzed using independent sample Student’s *t*-test (**C**–**F**,**H**,**I**,**K**–**M**). Error bars (**C**–**F**,**H**,**I**,**K**–**M**) show SEM. * *p* < 0.05, ** *p* < 0.01, *** *p* < 0.001. *p*-values less than 0.05 were considered statistically significant.

To further investigate whether the protective effect of an L-tryptophan-rich diet was in an AhR-dependent manner, mice were administered the AhR inhibitor CH223191. Mice were randomly assigned to four groups according to the method (Figure 6A). Reduced serum ALT and AST levels as well as aggravated inflammatory infiltration in the HIE+TRP5 group were notably abolished by the addition of the AhR inhibitor CH223191 (Figure 6B–D). Moreover, the effect of L-tryptophan supplementation attenuated macrophage aggregation in liver tissue and the level of TNF-α in serum in HIE mice, which could be inhibited by CH223191 (Figure 6E). Meanwhile, there was no significant difference in the mRNA expressions of the inflammatory cytokines TNF-α, IL-6, and IFN-γ (Figure 6F) between HIE and HIE+TRP5 with CH223191 in the liver. The decrease in chemokine mRNA levels (MCP-1, CXCL-1, CXCL-2, and CXCL-10) and the quantity of F4/80^+^ macrophages after L-tryptophan were also abolished by the CH223191 (Figure 6F–H). Interestingly, the CH223191 also counteracted the protective effects of the decrease in protein expression of IκBα and the ratio of phospho-p65 to p65 after L-tryptophan treatment (Figure 6J–L). Moreover, L-tryptophan treatment reduced the level of ROS in HIE-induced mice, while the protective effect could be attenuated by CH223191 (Figure 6G,I). Accordingly, the reversed effect of CAT, MDA, SOD, and GSH levels in the liver by L-tryptophan treatment was also abrogated by CH223191, respectively (Figure 6M). Overall, these results show that the protective effect of an L-tryptophan-rich diet against HIE-induced liver dysfunction and disturbed homeostasis is mediated through an AhR-dependent pathway.

### 3.6. IAA Intervention Improved LPS-Induced Hepatocyte Injury In Vitro and HIE-Induced Liver Dysfunction In Vivo

**Figure 7:**
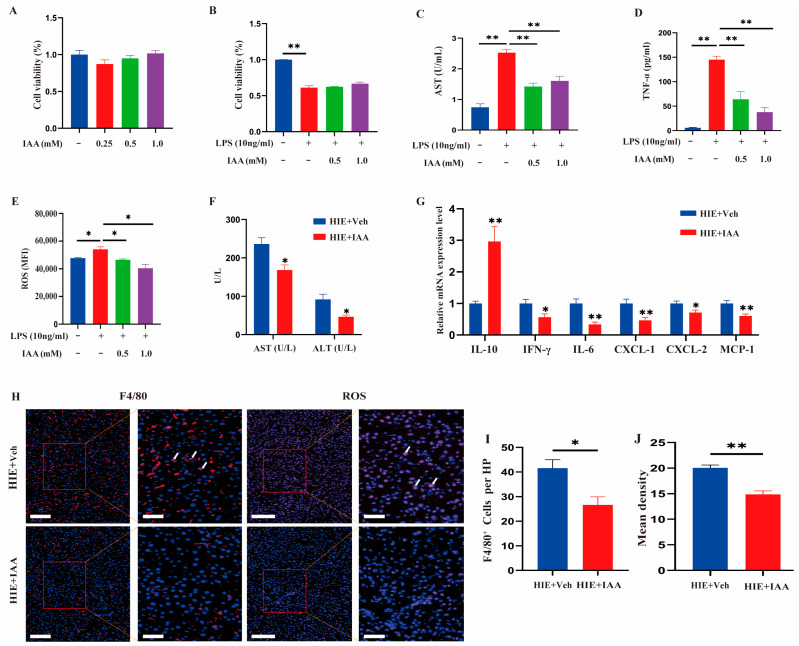
IAA intervention improved LPS-induced hepatocyte injury in vitro and HIE-induced liver dysfunction in vivo. (**A**) Cell viability of HepG2 cells treated with IAA of gradient concentrations (0, 0.25, 0.5, or 1.0 mM) for 24 h. (**B**–**E**) HepG2 cells were treated with or without IAA (0.5 or 1.0 mM) for 12 h, followed by LPS treatment for an additional 12 h. The cell viability (**B**), the supernatant AST (**C**) and TNF-α (**D**) levels, and ROS production by HepG2 cells (**E**) were detected. (**F**) Serum concentrations of ALT and AST of mice in HIE and HIE + IAA groups (n = 5). (**G**) The mRNA expression of IL-10, CXCL-1, CXCL-2, IFN-γ, IL-6, and MCP-1 in the liver of mice in the HIE and HIE + IAA groups analyzed using qRT-PCR (n = 5). (**H**) Fluorescence staining of macrophages in liver sections. F4/80^+^ macrophages, red with white arrow; nuclei, blue. Magnification, ×200; scale bars, 100 μm. Fluorescence staining of ROS production in liver sections (ROS, red with white arrow; nuclei, blue). Magnification ×200; scale bars, 100 μm. (**I**) Quantification of macrophages per high-power field (n = 3). (**J**) Quantification of ROS fluorescence intensity (n = 3). Data were analyzed using independent sample Student’s *t*-test (**A**–**G**,**I**,**J**). Error bars (**A**–**G**,**I**,**J**) show SEM. * *p* < 0.05, ** *p* < 0.01. *p*-values less than 0.05 were considered statistically significant.

To confirm whether tryptophan metabolite IAA could improve HIE-induced liver injury in vivo and in vitro, the LPS-induced HepG2 cells were established. The impact of IAA on HepG2 cell viability was detected through the CCK-8 assay. There was no notable difference in cell viability when the IAA was performed at concentrations of 0.25, 0.5, and 1.0 mM (Figure 7A). Furthermore, LPS treatment resulted in decreased cell viability in HepG2 cells, which could not be significantly inhibited by IAA treatment (Figure 7B). However, IAA treatment led to a significant inhibitory effect on LPS-induced decreased levels of AST and TNF-α in cell supernatants, as well as increased ROS levels in HepG2 cells (Figure 7C–E). Next, to evaluate the effect of IAA treatment on HIE-induced liver dysfunction, mice were randomly assigned to two groups: the HIE + vehicle and HIE +IAA groups. Compared with HIE + vehicle mice, the levels of AST and ALT in the serum were significantly reduced after IAA supplement (Figure 7F). To assess the effect of IAA on the expression of liver inflammatory cytokines, the mRNA levels of the pro-inflammatory and anti-inflammatory cytokines in the liver were examined. The mRNA expressions of IL-6 and IFN-γ were significantly decreased in the HIE + IAA group, compared with the HIE + vehicle group, whereas IAA treatment significantly elevated the mRNA level of IL-10 (Figure 7G). Meanwhile, we also found that the chemokine mRNA levels (MCP-1, CXCL-1, and CXCL-2) and the quantity of F4/80^+^ macrophages were significant reduced after IAA administration (Figure 7G–I). Particularly, the ROS levels were significantly decreased in the IAA treatment group compared with HIE + vehicle group mice (Figure 7H,J). These results indicated that the IAA intervention could improve LPS-induced hepatocyte injury in vitro and HIE-induced liver dysfunction in vivo.

## 4. Discussion

Regular physical activity has multifarious benefits for physical and mental health. Additionally, low- to moderate-intensity exercise training can result in the improvement of human health and mitigation of several chronic diseases. In addition to its beneficial effects, physical activity might also be associated with impaired physical health, being related to disturbances like “excessive exercise” and “overtraining syndrome”. Furthermore, excessive loading, insufficient recovery, and under preparedness might increase injury risks with several tissues and organs, including the musculoskeletal, cardiovascular, and digestive systems. This study demonstrated that HIE resulted in significant liver dysfunction, characterized by increased transaminase, inflammation, oxidative stress, glycogen exhaustion, and metabolic disturbance. Moreover, the nutritional metabolism disorder was associated with the occurrence and outcome of most patients with chronic liver diseases. The current research on amino acid supplementation mainly focuses on enhancing exercise performance and muscle recovery after HIE. For instance, the evidence-based information indicates that the use of BCAAs after exhaustive and damaging exercise is superior to passive recovery or rest, especially in terms of attenuating the loss of muscle strength and muscle power [16]. Moreover, studies have shown that the ingestion of a moderate dose of caffeine, leucine, and taurine before exercise enhances high-intensity exercise performance [17]. And tryptophan, an essential component of the human diet, was found to exert a notably protective effect against HIE-induced liver injury in our study for the first time. The tryptophan metabolite IAA was also found to be highly involved in the amelioration of HIE-induced liver dysfunction in vivo and LPS-induced hepatocyte dysfunction in vitro, which might be mediated through the activation of AhR. These findings provide valuable clues for a further focused study on HIE-induced liver function and the exploration of effective protection strategies.

The liver is the largest digestive organ and also a critical hub for numerous physiological processes, including macronutrient metabolism, blood volume regulation, immune system support, endocrine control of growth signaling pathways, lipid and cholesterol homeostasis, etc. Regular exercise can be considered as an effective strategy for treatment of chronic liver diseases such as non-alcoholic fatty liver disease (NAFLD). However, whether excessive or high-intensity exercise is either beneficial or detrimental to the liver has not been fully elucidated. Several forms of physical exercise, such as cycling and long-distance running, have been reported to be associated with postexercise increases in liver biomarkers associated with liver injury, particularly AST and ALT [18], which was also evidenced in our experiment. Particularly, our results showed that HIE led to significant metabolic disturbances in the liver, characterized by alterations to hundreds of metabolites. Through the biological classification of differential metabolites, we found that HIE led to the most pronounced increase in lipids and lipid-like molecules such as agatholic acid and lactulose. These metabolites may be closely related to the energy supply of the liver by promoting the decomposition of fatty acids and gluconeogenesis after exercise-induced carbohydrate consumption [19]. By analyzing the enriched KEGG pathways, we found that metabolic pathways related to glycogenic amino acids were highly involved. These pathways played a critical role, and the significant enhancement of gluconeogenesis may be an important way for the body to produce energy in addition to carbohydrates and fatty acids during HIE. Injury induced by exercise load manifests as inflammatory changes caused by unbalanced metabolic homeostasis. As the largest metabolic organ of the human body, the liver is prone to produce an inflammatory response to injury [20]. In our study, for the first time, we found that HIE can induce tryptophan metabolism disorder in the liver and serum, and the serum level of tryptophan showed a correlation with liver dysfunction, implying that tryptophan metabolism disorder may play a key role in HIE-induced liver dysfunction.

Nutritional metabolism was associated with the occurrence and prevention and treatment of liver injury in many diseases, which has attracted the attention of researchers [21]. Some reports have suggested that tryptophan affects various pathophysiological processes, including neuronal function, metabolism, inflammatory responses, oxidative stress, immune responses, and intestinal homeostasis [22]. Moreover, studies have found that tryptophan plays a protective role in some metabolic diseases of the liver, and that microbiota tryptophan metabolism improves alcohol-induced liver injury [23]. Our experiments found, for the first time, notable tryptophan metabolism disorder in HIE and the protective effect of an L-tryptophan-rich diet against liver dysfunction induced by HIE. L-tryptophan supplementation can also significantly increase blood glucose and hepatic glycogen levels, and the mechanism may be related to the elevation of hepatic glycogen synthase and hepatic glycogen phosphorylase. Simultaneous supplementation of L-tryptophan can reduce HIE-induced liver inflammation and oxidative stress, thereby attenuating liver dysfunction, which provides much evidence that adequate supplementation of tryptophan and a tryptophan-rich diet serves as an effective protective strategy against high-intensity exercise-induced liver injury.

After discovering that tryptophan can reduce liver damage caused by excessive exercise, we investigated how tryptophan works. Serum tryptophan was decreased after HIE but was not increased after L-tryptophan-rich diet supplementation, as measured by serologically targeted metabolomics. Furthermore, there was a significant increase in a series of tryptophan metabolites such as indole-3-propionic acid, indole-3-lactic acid, L-kynurenine, etc. The previous literature has confirmed that IPA improved the intestinal barrier by enhancing the epithelial barrier and mucus barrier [24]. IAA supplementation restored intestinal levels in mice and prevented ethanol-induced steatohepatitis by inducing intestinal expression of IL-22 and Reg3γ, thereby preventing bacterial transfer to the liver [25]. We then performed a correlation analysis and found that IAA was negatively correlated with the serum AST level, implying that IAA may play an important role in alleviating HIE-induced liver injury in L-tryptophan supplementation. Furthermore, we speculate that IAA may exert protective effects by active AhR performing molecular docking. Meanwhile, we found that IAA increased the AhR and CYP1A1 mRNA levels in the liver. Therefore, in our study, firstly, we demonstrated that the protective effect of an L-tryptophan-rich diet was indeed abolished by the AhR inhibitor, and we next found that IAA supplementation could improve HIE-induced liver dysfunction. IAA might have other pathways to mitigate the liver injury induced by HIE. For instance, IAA metabolized by the gut microbiota maintains homeostasis of the intestinal epithelium through mucin sulfation [26]. The integrity of the intestinal barrier can prevent toxic substances from harming the liver. Further studies are needed to confirm this hypothesis.

Thus, we proposed that an L-tryptophan-rich diet improves high-intensity-exercise-induced liver dysfunction via the metabolite indole-3-acetic acid and AhR activation. There were still some limitations in our research. The CH223191 inhibitor is a systemic AhR inhibitor rather than a liver-specific AhR inhibitor. We discovered that a tryptophan-rich diet alleviates high-intensity-exercise-induced liver dysfunction. However, there are two aspects of the problem that restrict the development of tryptophan supplements. One is that the protective effects of TRP not only operate through the tryptophan prototype but also through its metabolites, such as IAA intervention improving HIE-induced liver dysfunction. More research on tryptophan and its metabolites is necessary to assist in enhancing the efficacy of supplements in organisms. Additionally, due to the numerous metabolites of tryptophan in the body, tryptophan supplementation may have certain side effects. Studies have indicated that serotonin, a metabolite of tryptophan, is associated with the development of depression [27]. Moreover, gut-microbiota-derived tryptophan metabolism mediates renal fibrosis through aryl hydrocarbon receptor signaling activation [28]. Therefore, more research is required to reduce the side effects and increase the efficacy of tryptophan supplements. To address this issue, mice with a specific knockout of AhR in the liver are needed.

## 5. Conclusions

We demonstrated that HIE induces significant metabolic disorders and liver dysfunction. An L-tryptophan-rich diet (0.5% tryptophan *w/w*) significantly attenuated excessive exercise-induced liver dysfunction. Combining with tryptophan targeted metabolomics and molecular docking, we found that the mechanism in dietary tryptophan supplementation was via increased tryptophan metabolite IAA and AhR activation. Furthermore, we demonstrated that the protective effect of an L-tryptophan-rich diet was indeed abolished by the AhR inhibitor and IAA intervention can protect from excessive exercise-induced liver dysfunction in vivo and LPS-induced hepatocyte injury in vitro. Together, an L-tryptophan-rich diet can activate high-intensity exercise-induced liver dysfunction via the metabolite indole-3-acetic acid and AhR activation. 

## Figures and Tables

**Table 1:** Sequences of primers used in qRT-PCR.

Target Gene	Primers (5′→3′)
*TNF-α*	F: 5′-ATGTCTCAGCCTCTTCTCATTC-3′
	R: 5′-GCTTGTCACTCGAATTTTGAGA-3′
*IL-6*	F: 5′-CTCCCAACAGACCTGTCTATAC-3′
	R: 5′-CCATTGCACAACTCTTTTCTCA-3′
*IL-10*	F: 5′-GCTCCAAGACCAAGGTGTCT-3′
	R: 5′-CGGAGAGAGGTACAAACGAGG-3′
*CXCL-1*	F: 5′-CGCCTATCGCCAATGAGC-3′
	R: 5′-AGCTTCAGGGTCAAGGCAA-3′
*CXCL-2*	F: 5′-GAAGTCATAGCCACTCTCAAGG-3′
	R: 5′-CCTCCTTTCCAGGTCAGTTAGC-3′
*CXCL-10*	F: 5′-GAAATTATTCCTGCAAGCCAATTT-3′
	R: 5′-TCACCCTTCTTTTTCATTGTAGCA-3′
*MCP-1*	F: 5′-TCCCAATGAGTAGGCTGGAG-3′
	R: 5′-TCTGGACCCATTCCTTCTTG-3′
*IFN-γ*	F: 5′-CTGGAGGAACTGGCAAAAGGATGG-3′
	R: 5′-GACGCTTATGTTGTTGCTGATGGC-3′
*β-actin*	F: 5′-CTACCTCATGAAGATCCTGACC-3′
	R: 5′-CACAGCTTCTCTTTGATGTCAC-3′

*β-actin*, actin, beta; *TNF-α*, tumor necrosis factor-α; *IL-6*, interleukin-6; *IL-10*, interleukin 10; *CXCL-1*, *C-X-C* motif chemokine ligand-1; *CXCL-2*, C-X-C motif chemokine ligand-2; *CXCL-10*, C-X-C motif chemokine ligand-10; *MCP-1*, monocyte chemoattractant proteins-1; *IFN-γ*, interferon-gamma.

**Table 2:** Differential metabolites of liver between HIE and CON group mice.

Superclass	Name	Class	Subclass	VIP	Fold Change	*p*-Value
	Beta-hydroxybutyrate	Hydroxy acids and derivatives	Beta hydroxy acids and derivatives	2.81	4.26	<0.001
	Malonic acid	Carboxylic acids and derivatives	Dicarboxylic acids and derivatives	1.63	0.36	<0.001
	Ser-Pro-Arg	Carboxylic acids and derivatives	Amino acids, peptides, and analogues	1.64	0.34	<0.001
	Pro-hyp	Carboxylic acids and derivatives	Amino acids, peptides, and analogues	1.25	0.49	<0.001
Organic acids	Carbamoylaspartate	Carboxylic acids and derivatives	Amino acids, peptides, and analogues	1.30	0.31	<0.001
and derivatives	Asparagine	Carboxylic acids and derivatives	Amino acids, peptides, and analogues	2.31	0.47	<0.001
	Lys-Val	Carboxylic acids and derivatives	Amino acids, peptides, and analogues	1.11	2.41	<0.001
	Histidine	Carboxylic acids and derivatives	Amino acids, peptides, and analogues	8.33	0.41	<0.001
	L-thyronine	Carboxylic acids and derivatives	Amino acids, peptides, and analogues	3.29	0.27	<0.001
	Jasplakinolide	Peptidomimetics	Hybrid peptides	2.96	0.27	<0.001
	Lactulose	Prenol lipids	Diterpenoids	1.82	0.23	<0.001
	D-Maltose	Fatty Acyls	Fatty acyl glycosides	1.80	0.23	<0.001
	L-palmitoylcarnitine	Glycerophospholipids	Glycerophosphoinositol phosphates	2.71	4.36	<0.001
Organic acids	Myristoleic acid	Fatty Acyls	Fatty acid esters	2.19	4.46	<0.001
and derivatives	Isomaltose	Fatty Acyls	Fatty acids and conjugates	6.67	0.32	<0.001
	1-hexadecyl-sn-glycero-3-phosphocholine	Glycerophospholipids	Glycerophosphoinositol phosphates	3.49	3.44	<0.001
	Tetradecanedioic acid	Glycerophospholipids	Glycerophosphocholines	1.25	4.10	<0.001
	Aminocaproic acid	Fatty Acyls	Fatty acids and conjugates	1.03	0.50	<0.001
	1-(1,2-dihexanoylphosphatidyl)inositol-4-phosphate	Fatty Acyls	Fatty acyl glycosides	2.62	0.19	<0.001
	1-(1,2r-dioctanoylphosphatidyl)inositol-3,4-bisphosphate	Fatty Acyls	Fatty acyl glycosides	2.26	0.24	<0.001
	Humulone	Organooxygen compounds	Carbonyl compounds	3.39	9.88	<0.001
	D-Glucuronate	Organooxygen compounds	Carbohydrates and carbohydrate conjugates	10.17	0.34	<0.001
	L-Sorbose	Organooxygen compounds	Carbohydrates and carbohydrate conjugates	1.15	0.26	<0.001
	Stachyose	Organooxygen compounds	Carbohydrates and carbohydrate conjugates	2.11	0.33	<0.001
Organic oxygen	Maltotetraose	Organooxygen compounds	Carbohydrates and carbohydrate conjugates	12.53	0.32	<0.001
compounds	Glutaraldehyde	Organooxygen compounds	Carbonyl compounds	1.45	0.44	<0.001
	Ribitol	Organooxygen compounds	Carbohydrates and carbohydrate conjugates	1.66	0.27	<0.001
	Lactose	Organooxygen compounds	Carbohydrates and carbohydrate conjugates	2.86	0.36	<0.001
	Tubuloside a	Organooxygen compounds	Carbohydrates and carbohydrate conjugates	2.49	0.28	<0.001
	D-psicose	Organooxygen compounds	Carbohydrates and carbohydrate conjugates	1.42	0.44	<0.001
	D-Glucuronolactone	Lactones	Gamma butyrolactones	1.95	0.33	<0.001
	Dihydrouracil	Diazines	Pyrimidines and pyrimidine derivatives	3.30	0.39	<0.001
	4-pyridoxic acid	Pyridines and derivatives	Pyridinecarboxylic acids and derivatives	2.18	0.56	<0.001
	Thiamine	Diazines	Pyrimidines and pyrimidine derivatives	5.62	0.57	<0.001
Organoheterocy	Ethoxyquin	Quinolines and derivatives	Quinolones and derivatives	1.84	0.07	<0.001
clic compounds	Enrofloxacin	Quinolines and derivatives	Quinoline carboxylic acids	1.57	0.29	<0.001
	Pheniramine	Pyridines and derivatives	Pheniramines	1.28	1.86	<0.001
	L-gulono-1,4-lactone	Lactones	Gamma butyrolactones	4.17	0.59	<0.001
	Cytosine	Diazines	Pyrimidines and pyrimidine derivatives	3.73	0.55	<0.001

**Table 3:** Correlation analysis of metabolites and AST or ALT (* *p* < 0.05).

Name	AST		ALT	
	R	*p*-Value	R	*p*-Value
Indoleacetate	−0.6504	0.022 *	−0.5467	0.0659
L-kynurenine	−0.5039	0.0949	−0.3307	0.2937
Indole-3-propionic acid	−0.4644	0.1282	−0.5568	0.0601
Indole-3-lactic acid	−0.5059	0.0933	−0.1851	0.5647
Xanthurenate	−0.5033	0.0953	0.08369	0.7959
N-formyl-kynurenine	−0.3793	0.224	−0.4298	0.1632
3-hydroxyl-L-kynurenine	−0.3308	0.2937	0.01773	0.9564

## Data Availability

The datasets used and/or analyzed during the current study are available from the corresponding author on reasonable request.

## Supplementary Materials

The following supporting information can be downloaded at https://www.mdpi.com/article/10.3390/cells14080605/s1: Table S1: Correlation analysis between metabolites and AST or ALT. Figure S1: Tryptophan supplement changed HIE-induced tryptophan metabolism by targeted metabolomics. The serum concentration of tryptophan metabolites tested by targeted metabonomics in mice of HIE and HIE + TRP5 groups (A–Q). Data were analyzed by independent sample student’s *t*-test. Error bars show SEM. * *p* <0.05, ** *p* <0.01, *** *p* <0.001; Figure S2: the schematic diagram of the experimental designs. In figure A, we have demonstrated the design of experience 1–4. the intervenions of all CON and HIE groups are following by figure B. Figure S3: Western blot quantifications in experiments.

## Abbreviations

AhR, aryl hydrocarbon receptor; ALT, alanine transaminase; AST, aspartate aminotransferase; CAT, catalase; CXCL-1, C-X-C motif chemokine ligand-1; CXCL-2, C-X-C motif chemokine ligand-2; CXCL-10, C-X-C motif chemokine ligand-10; DSS, dextran sulfate sodium; GSH; glutathione; HIE, high-intensity exercise; H&E, hematoxylin and eosin; IAA, indole-3-acetic acid; IFN-γ, interferon-gamma; IL-6, interleukin-6; IL-10, interleukin-10; IPA, indole-3-propionic acid; MCP-1, monocyte chemoattractant proteins-1; MDA, malonaldehyde; NAFLD, non-alcoholic fatty liver disease; NASH, nonalcoholic steatohepatitis; PAS, periodic acid-Schiff; PCA, principal component analysis; qRT-PCR, quantitative real-time polymerase chain reaction; ROS, reactive oxygen species; REG3G, regenerating islet-derived 3 gamma; SOD, superoxide dismutase; TNF-α, tumor necrosis factor-α; VO_2_ max, maximal oxygen consumption.
