# Supplementary material for: L-Tryptophan-Rich Diet Alleviates High-Intensity-Exercise-Induced Liver Dysfunction via the Metabolite Indole-3-Acetic Acid and AhR Activation
Source: Cells. 2025 Apr 16;14(8):605. doi: 10.3390/cells14080605 (PMC12026455; doi:10.3390/cells14080605)
Supplement: Supplementary file 1 [file cells-14-00605-s001.zip › Table S1.pdf]

Table S1. Correlation analysis between metabolites and AST or ALT

Name	AST		ALT	
	R	p-value	R	p-value
Indoleacetate	-0.6504	0.022*	-0.5467	0.0659
L-kynurenine	-0.5039	0.0949	-0.3307	0.2937
Indole-3-propionic acid	-0.4644	0.1282	-0.5568	0.0601
Indole-3-lactic acid	-0.5059	0.0933	-0.1851	0.5647
Xanthurenate	-0.5033	0.0953	0.08369	0.7959
N-formyl-kynurenine	-0.3793	0.224	-0.4298	0.1632
3-hydroxyl-L-kynurenine	-0.3308	0.2937	0.01773	0.9564
Picolinic acid	0.1322	0.6011	0.5219	0.0263
5-Hydroxyindole-3-acetic acid	-0.3471	0.1582	-0.2227	0.3745
Cinnavalininate	0.1635	0.5169	0.02282	0.9284
kynurenate	-0.3831	0.1166	-0.2002	0.4257
serotonin	0.1473	0.5597	-0.05324	0.8338
3-Indoxyl sulfate	0.1994	0.4277	0.06541	0.7965
Indoxyl-b-D-glucuronide	-0.02092	0.9343	-0.2209	0.3784
Quinolinic acid	-0.2116	0.3992	0.1657	0.5111
