# Supplementary figures and images for: L-Tryptophan-Rich Diet Alleviates High-Intensity-Exercise-Induced Liver Dysfunction via the Metabolite Indole-3-Acetic Acid and AhR Activation
Source: Cells. 2025 Apr 16;14(8):605. doi: 10.3390/cells14080605 (PMC12026455; doi:10.3390/cells14080605)

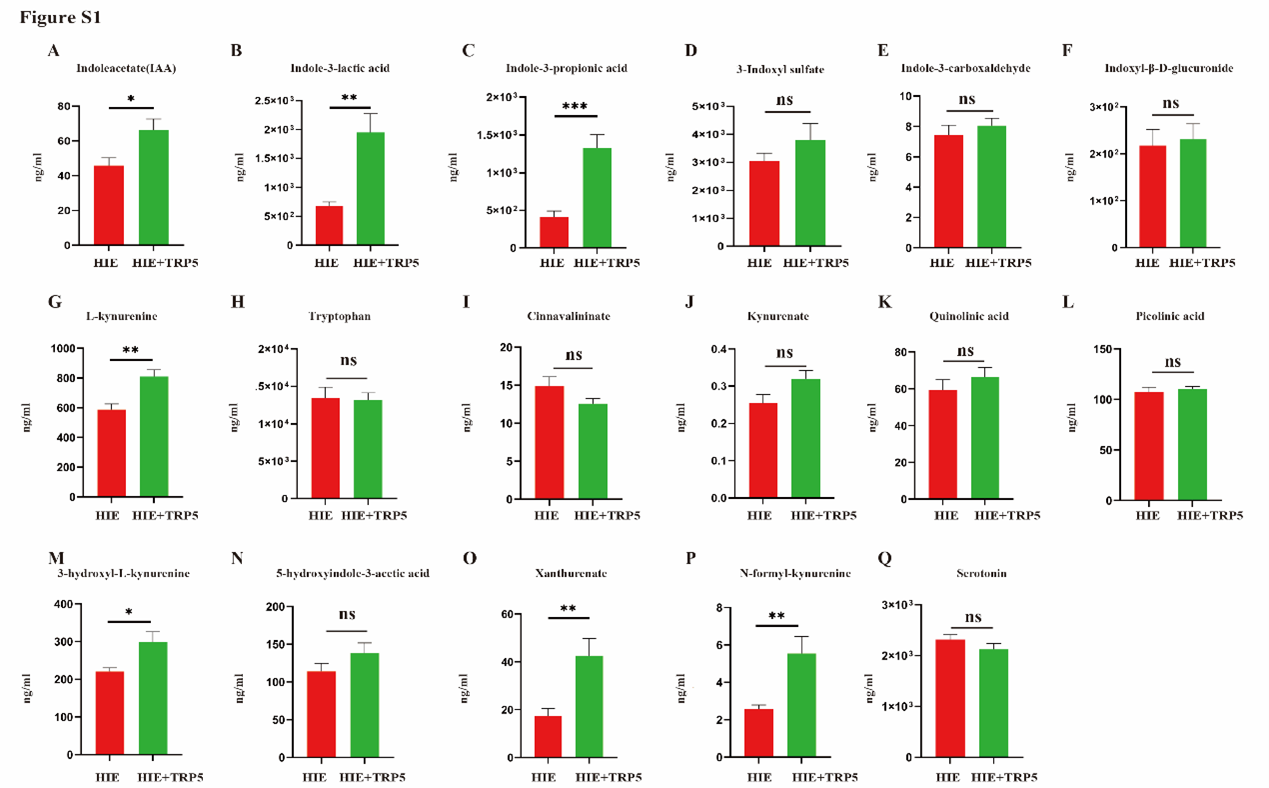

Supplement: Supplementary file 1 [file cells-14-00605-s001.zip › Supplementary Figure/Figure S1.tif]

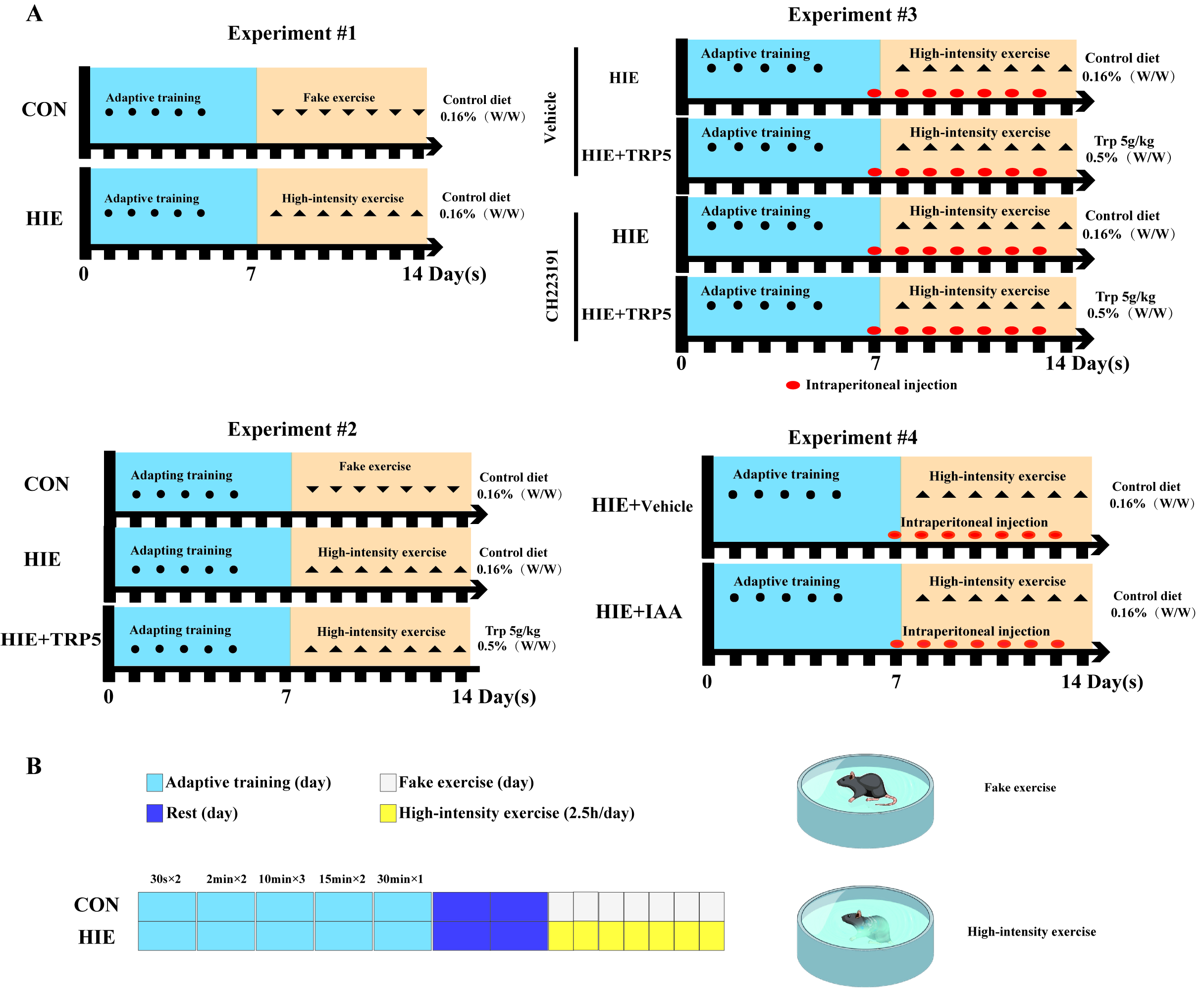

Supplement: Supplementary file 1 [file cells-14-00605-s001.zip › Supplementary Figure/Supplementary Figure S2.tif]

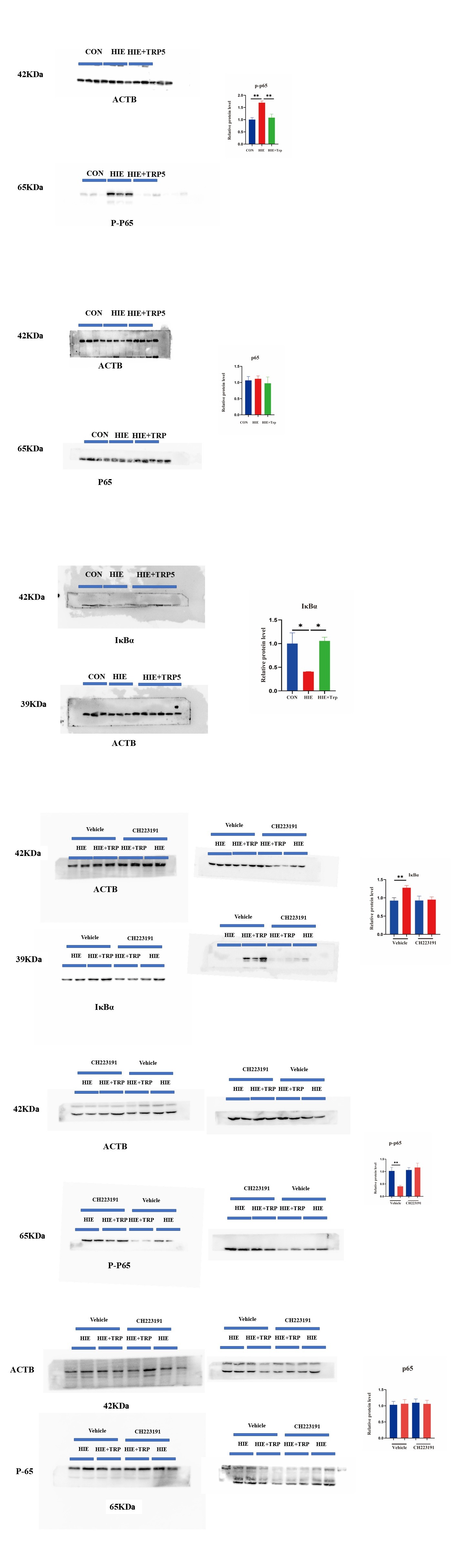

Supplement: Supplementary file 1 [file cells-14-00605-s001.zip › Supplementary Figure/Supplementary figure S3.jpeg]
